# Advanced Cell Culture Techniques for Cancer Drug Discovery

**DOI:** 10.3390/biology3020345

**Published:** 2014-05-30

**Authors:** Carrie J. Lovitt, Todd B. Shelper, Vicky M. Avery

**Affiliations:** Discovery Biology, Griffith University, N27, Don Young Road, Nathan, Queensland, 4111, Australia; E-Mails: carrie.lovitt@griffithuni.edu.au (C.J.L.); t.shelper@griffith.edu.au (T.B.S.)

**Keywords:** 3D culture, microenvironment, drug resistance, tumor models, high-throughput screening

## Abstract

Human cancer cell lines are an integral part of drug discovery practices. However, modeling the complexity of cancer utilizing these cell lines on standard plastic substrata, does not accurately represent the tumor microenvironment. Research into developing advanced tumor cell culture models in a three-dimensional (3D) architecture that more prescisely characterizes the disease state have been undertaken by a number of laboratories around the world. These 3D cell culture models are particularly beneficial for investigating mechanistic processes and drug resistance in tumor cells. In addition, a range of molecular mechanisms deconstructed by studying cancer cells in 3D models suggest that tumor cells cultured in two-dimensional monolayer conditions do not respond to cancer therapeutics/compounds in a similar manner. Recent studies have demonstrated the potential of utilizing 3D cell culture models in drug discovery programs; however, it is evident that further research is required for the development of more complex models that incorporate the majority of the cellular and physical properties of a tumor.

## 1. Introduction

Historically, the only procedures developed for whole cell-based screening assays were those utilizing a flat layer of cells attaching to various plastic substrata. However, it is now generally accepted that culturing cells in these two-dimensional (2D) conditions is not physiologically relevant, and difficulties may be encountered downstream with translation *in vivo*. The tissue-specific architecture, along with elements in the surrounding microenvironment, are essential components of a tumor and can be at least partially recapitulated utilizing three-dimensional (3D) cell culture models [1,2].

High-throughput screening (HTS) using cell-based assays has frequently been the starting point for identifying novel compounds in drug discovery programmes. The procedures in place for the development of drugs involve thorough evaluation of novel drug candidates in both pre-clinical and clinical phases. Through these drug development practices, the attrition rates of drug candidates for cancer are significant, being approximately 95% [3]. The development of more biologically relevant *in vitro* tumor models may ultimately result in improved translation and a reduction in number of the animal models required in drug discovery programmes [4].

This review focuses on the culturing of cell lines representative of solid cancers in advanced cell culture conditions. We discuss the molecular aspects of cells cultured in 3D and their relevance to cancer, focusing on key examples from the literature. We will also examine the 3D models that have been successfully implemented in early stage compound screening and the future of *in vitro* cell-based assays in cancer drug discovery practices.

## 2. Modeling Cancer in 3D Cell Culture

**Figure 1:**
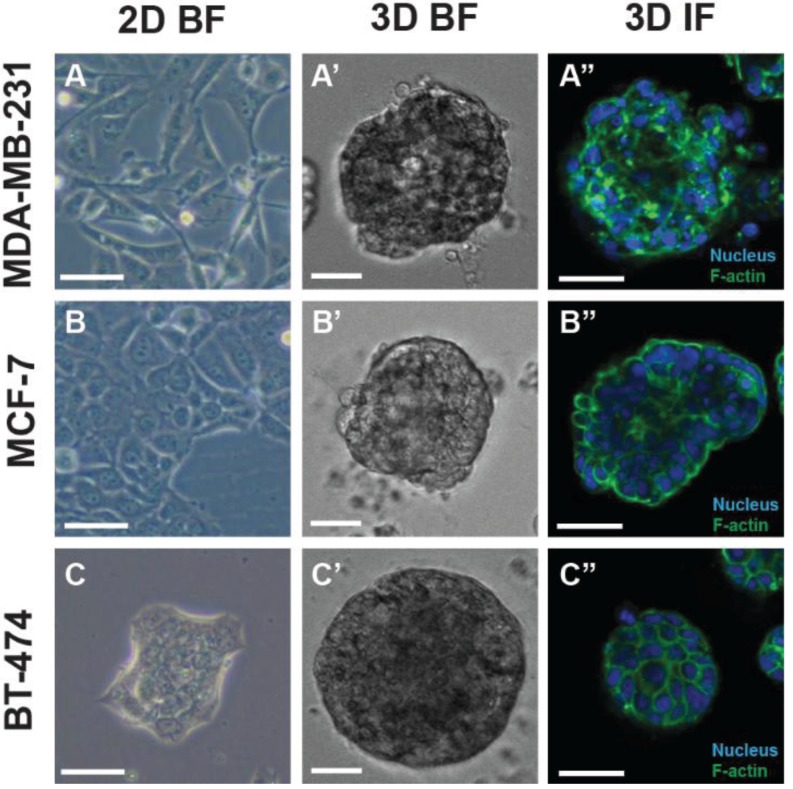
Phenotypic properties of a panel of breast cancer cell lines cultured in two-dimensional (2D) and three-dimensional (3D) cell culture systems. Brightfield (BF) and immunofluorescence (IF; central Z-slice through a spheroid) microscopy illustrate 2D cell cultures and 3D structures unique to each cell line. MDA-MB-231 in 2D (**A**) and 3D (**A’**, **A’’**), MCF-7 in 2D (**B**) and 3D (**B’**, **B’’**) and BT-474 in 2D (**C**) and 3D (**C’**, **C’’**). Scale bar = 50 μm.

A range of 3D cell culture techniques have been developed, which can be applied to various research applications including cancer drug discovery. However, there are differing interpretations of what culturing in the third dimension actually means. For the purposes of this review, the term shall be used in reference to cells assembled into 3D structures which are cultured using either anchorage-independent methodology (without the use of a substrate for cellular attachment) or anchorage-dependent conditions (utilizing a substrate which promotes cellular attachment). The phenotypic characteristics of cancer cells cultured in a 3D model are evident in comparison to cells grown as planar cultures (Figure 1). 

Anchorage-independent 3D cell culture methods involve the aggregation of cells in non-adherent culture conditions where there is no substrate, such as extracellular matrix (ECM) proteins available for cellular attachment. This 3D cell culture method can be achieved by using low-attachment plates [5] and through coating surfaces, for example with poly-hydroxyethyl methacrylate (poly-HEMA) [6] or agarose [7]. Another approach is the hanging drop method, where a drop of media containing a cell suspension promotes cell-to-cell interactions within the confines of the drop [8]. 3D cultures can also be generated in an anchorage-independent manner by culturing cells with soft agar [9]. An additional anchorage-independent 3D environment can be established with the use of pre-fabricated scaffolds, which consist of porous materials to support the growth of 3D structures [10]. Furthermore, spheroids can be created as a result of agitation procedures such as spinner flasks or a gyratory shaker [11]. The above-mentioned approaches generate types of spheroids which are commonly referred to as multicellular tumor spheroids (MCTS) in cancer research. These spheroids may exhibit tumor-specific characteristics such as heterogeneous proliferation rates, nutrient and oxygen gradients, a central region of necrosis as well as cell-to-cell and ECM-to-cell contacts in a 3D context [12,13,14]. 

In addition to the anchorage-independent model, the formation of anchorage-dependent 3D cell structures resulting from cells adhering to specific substrates have been developed. One of these specialized substrates is comprised of a membrane, and the resultant structures are commonly referred to as multilayered cell cultures (MCCs). MCCs are composed of tumor cells cultured on a membrane and are specifically designed to allow measurement of drug diffusion [15,16]. Microfluidics channels which employ micropillars are also able to support the formation of 3D cell cultures and, in addition, ECM can also be added into these chambers to allow ECM-to-cell interactions [17]. Basement membrane extract from the Engelbreth-Holm-Swarm murine tumor, a form of laminin-rich ECM (lrECM), has been extensively utilized to promote the growth of cancer cells in 3D structures [18,19,20,21]. In addition to cancer research, lrECM has been employed as a biologically relevant scaffold for the elucidation of *in vivo* functional processes of non-malignant tissue *in vitro* [22,23,24,25]. The methods for culturing cells in 3D utilizing lrECM as a subtrate involves seeding a single cell suspension either on top of matrices (3D ‘on top’ assay) or mixed into lrECM (3D ‘embedded’ assay), which promotes the formation of cells into 3D structures in a time-dependent manner [26]. LrECM is not the only biologically relevant matrix available for 3D cell culture. Collagen I has also been utilized as a substrate for culturing tumor cells in 3D systems [27]. Malignant cells cultured in 3D utilizing lrECM as a substrate display distinctive morphologies [28,29], which is in contrast to the more uniform cellular aggregation observed in anchorage-independent 3D cell culture [7,30]. There are advantages and disadvantages associated with utilizing either anchorage-dependent or anchorage-independent 3D tumor models, which have been reviewed in detail elsewhere [31]. 

## 3. Drug Resistance and 3D Tumor Models

**Figure 2:**
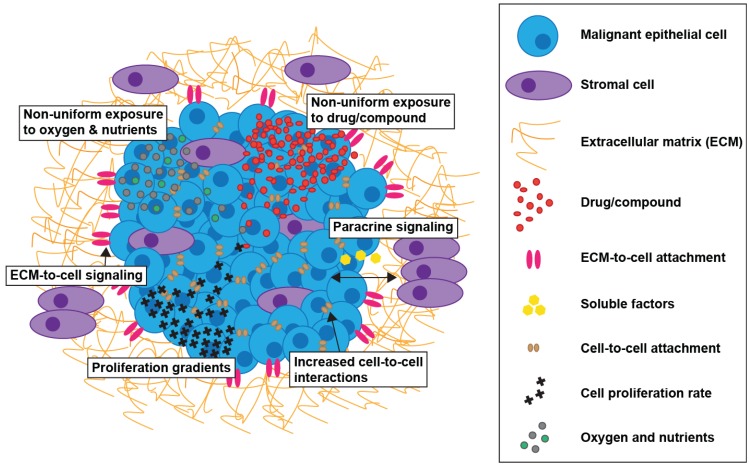
Advantages of incorporating elements of the tumor microenvironment in drug discovery programmes.

Drug resistance in cancer can be mediated by two different mechanisms, namely acquired and *de novo*. Acquired resistance results from modifications which occur during exposure of tumors to therapeutics and *de novo* resistance is associated with factors, such as adhesion of tumor cells to ECM, that existed prior to therapy [32,33]. The resistance mechanisms implicated in reducing drug effectiveness when the cancer patient is undergoing therapy include non-specific mechanisms, such as increased drug efflux from tumor cells, specific cellular processes of down-regulation or up-regulation of a drug target, the presence of cancer stem cells and the influence of tumor microenvironmental components [34]. Research suggests that solid tumors adapt quickly to treatment with chemotherapeutics, with genomic alterations detected shortly after cellular exposure to drugs [35]. A number of factors affecting the activity of anti-cancer drugs *in vivo* are able to be recreated *in vitro* utilizing 3D cell culture models [36]. The advantages of exploiting cells grown in 3D culture conditions in comparison to 2D culture models for evaluating drug candidates and exploring mechanistic properties of anti-cancer agents can include: (i) oxygen and nutrient gradients, (ii) increased cell-to-cell interactions resulting from cellular formation into 3D architecture, (iii) non-uniform exposure of cells within a spheroid to drug/compound, (iv) ECM-to-cell signaling, (v) different rates of cellular proliferation throughout the 3D structure and, (vi) impact of stromal/tumor site specific cells in the tumor microenvironment (Figure 2). 

Numerous anti-cancer therapeutics have been evaluated in a range of different cancer cell types in 3D cell culture systems and directly compared to the same cells grown in a 2D monolayer format. Studies have have shown that tumor cells were less sensitive to anti-cancer agents when evaulated in a 3D system compared to 2D culture conditions [37,38,39]. However, there has also been a number of studies where the observed effects of anti-cancer agents against tumor cells in 3D culture were equal to, or more sensitive to, the same tumor cell type cultured in a 2D monolayer format [40,41,42]. The information acquired from employing these 3D cell cultures in cancer research, irrespective of whether the cellular sensitivity to drugs/compounds is increased or decreased, has the potential to provide a more accurate representation of drug/compound activity *in vivo*. For instance, if the drug/compound sensitivity is decreased in tumor cells cultured in a 3D model, what mechanism/s of resistance are in play and how could these parameters affect the anti-tumor action of the drug/compound *in vivo*? Alternatively, if drug/compound sensitivity against tumor cells is increased in 3D cultures, is there a greater dependence on the target in the 3D cellular system? Or, are there indirect influences on drug/compound activity in tumor cells propagated in 3D culture not observed against cells cultured in 2D monolayers? The development of more advanced 3D cell culture models, such as those incorporating components from the tumor microenvironment, will be valuable in deconstructing these mechanistic elements.

## 4. Cellular Signaling in 3D Tumor Models

Culturing cells in 3D was envisaged decades ago as having potential for use in functional studies of malignant and/or non-malignant tissue [22,43,44,45,46]. In more recent years, an explosion of new techniques and the extensive characterization of a wide range of cancer cell lines has given researchers the opportunity to dissect cellular pathways in these more biologically relevant models and, in some instances, make comparative assessments to cells in traditional 2D monolayer culture. If pathways of cells in 2D models are not representative of the *in vivo* microenvironment, then screening for active compounds utilizing these models may not be as predictive. For example, the cellular target of a particular compound may not be expressed in the same quantities *in vivo* or the cellular signaling occuring *in vivo* may not be reflected in cells grown as 2D monolayers and therefore impact on the outcome.

A significant volume of research into molecular alterations of cells has been performed utilizing 3D cancer models, including examination of the genetic variations between different cell culture conditions. In one study, the gene expression of a panel of 24 malignant and non-malignant breast cell lines was compared between 2D monolayer cultures and 3D cell cultures generated on lrECM [28]. Significant modifications in gene expression were detected for genes encoding signal transduction proteins across the panel of cell lines tested, which provides supporting evidence that cellular signaling is altered in 3D cell cultures established on lrECM. In addition, changes in gene expression were also examined in a panel of malignant and non-malignant prostate cell lines [29]. In the 3D cell cultures of these cell lines, the gene expression patterns reflected the decreased cellular propagation upon culturing cells in lrECM in comparison to that of cells in 2D monolayer culture. Furthermore, research into changes in gene expression occurring between 2D and 3D cell cultures has also been completed in melanoma cells. A study by Ghosh *et al.* [47] demonstrated that 106 genes were up-regulated and 73 genes down-regulated in anchorage-independent 3D cell culture in comparison to the 2D monolayer cell cultures for the same cell line (NA8). The genetic alterations of interest included a number of chemokines (CXCL1, CXCL2 and CXCL3), IL-8 and CCL20 which were significantly up-regulated in cells cultured in 3D conditions.

Studies have also investigated specific alterations at the protein level of cells cultured in 3D systems. In a large panel of ovarian cancer cell lines, modifications in cell adhesion marker expression were observed, particularly for vimentin and E-cadherin, in 3D cell cultures when directly compared to 2D monolayer cell cultures [48]. In addition, a proteome analysis between 2D and 3D cell cultures of the colon cancer cell lines COGA-5 and COGA-12 was undertaken [49]. Results demonstrated alternative protein expression of certain proteins, for example, hydroxyprostaglandin dehydrogenase and lamin A/C in GOGA-5 cells cultured as spheroids in comparison to the same cells grown in 2D monolayer cultures. A 3D co-culture model for prostate cancer was developed to evaluate interactions between prostate cancer cells and stromal cells derived from the bone [50]. The prostate cancer cell line, PC3, was co-cultured with the bone stromal cell line, HS5, in 3D cell cultures generated on lrECM. In these co-cultures, cell-to-cell interactions and cross-talk between the different cell types was demonstrated, with the re-expression of CXCR7 and N-cadherin occuring in HS5 cells. Furthermore, α6- and/or β1-integrins were shown to influence the expression of certain cellular components, for example, E-cadherin and vimentin not only in 3D co-culture conditions, but also in mono-culture of PC3 and HS5 cells. It is evident that culturing cells in 3D alters gene and protein expression, however, it is yet to be determined if any of the changes observed between in tumor cells of 2D and 3D cell cultures will lead to identification of novel drug targets and if the changes in expression alone can influence the sensitivity of anti-cancer drugs.

The presence of ECM in the tumor microenvironment has been proven to effect drug activity against a variety of cancer cells. Numerous studies, in a range of different cancer cell lines, have shown that cancer cells cultured in 2D monolayers in the presence of different components of ECM proteins have reduced sensitivity to anti-cancer agents. For instance, in the breast cancer cell line MDA-MB-231, adhesion of α5β1- and α2β1-integrin to fibronectin and collagen I, respectively, was protective against paclitaxel cytotoxicity [51]. In lung cancer, a panel of cell lines (H69, H345 and H510) were cultured on either collagen IV, laminin or fibronectin and exposed to doxorubicin, cyclophosphamide or etiposide, with cellular attachment to all substrates resulting in increased cellular viability upon application of these apoptotic stimuli [52]. Furthermore, pancreatic cancer cells (AsPc‑1) cultured on laminin were partially protected from gemcitabine, with the signaling of focal adhesion kinase (FAK) demonstrated to be a contributing factor [53]. Cellular adhesion to ECM has also affected the sensitivity of anti-cancer agents on cells in 2D monolayer culture of prostate cancer [54] and glioblastoma cells [55]. Thus, attachment of tumor cells to specific ECM proteins affect the response of a wide range of cancer cells to therapeutics in 2D monolayer cell culture.

The 3D architecture of spheroids, in addition to the presence of ECM proteins in cell culture models, affect cellular responses to chemotherapeutic drugs. A study undertaken by Hakanson *et al.* [56] demonstrated this concept, revealing that a small 3D structure (less than 6 cells per aggregate) consisting of MCF-7 breast cancer cells was more resistant to paclitaxel in the presence of ECM proteins when compared to the same cells cultured in 2D on a layer of identical ECM proteins. Spheroid models have been ulilized to evaulate tumor cell signaling in comparison to 2D monolayer cultures in various cell lines. Notably, signaling downstream from human epidermal growth factor receptor type (Her2) was altered when cancer cells were cultured in anchorage-independent 3D conditions in comparison to a 2D monolayer format [42]. Specifically, a switch from the phosphoinositide 3-kinase (PI3K) pathway to the mitogen activated kinase (MAPK) pathway was demonstrated in breast, lung and ovarian cancer cell lines. The cell culture condition-dependent pathway switch was also observed in a study undertaken by Weigelt *et al.* [57] using breast cancer cell lines cultured in an lrECM-based model. 

Studies into the activity of anti-cancer agents against cells cultured as anchorage-independent spheroids have been performed. Mesothelioma cancer cell lines (M28, REN and VAMT) cultured in both 2D culture and a 3D anchorage-independent cell culture format exhibited resistance in the 3D system in response to application of apoptotic stimuli [39]. Certain proteins from the PI3K pathway were identified as having a role in mediating the observed resistance. Furthermore, decreased doxorubicin activity was detected in selected endometrial cancer cell lines cultured as anchorage-independent spheroids in comparison to the same cells cultured in 2D monolayers [58]. The enhanced resistance was potentially associated with cell line-dependent mechanisms including altered signaling through the PI3K pathway and altered antioxidant protein presence. Variations in drug activity against cells cultured as anchorage-independent spheroids compared to the same cells grown in 2D monolayer cultures is not unique to the types of cancer mentioned above. Altered drug sensitivity in cells cultured as anchorage-independent spheroids compared to cells cultured in a monolayer has also been observed in bladder [59], pancreatic [60,61] and colon cancer [62].

Altered signaling and sensitivity to anti-cancer agents was also observed in cell lines when cultured as 3D structures using lrECM as a substrate. The susceptibility of breast cancer cell lines over-expressing Her2 (AU565, SKBR3, HCC1569) to therapeutics targeting Her2 signaling (trastuzumab, pertuzumab and lapatinib) was cell line-, cell culture condition (2D *vs.* 3D)- and drug-dependent [57]. For instance, SKBR3 cells were significantly more resistant, AU565 cells were significantly less resistant and HCC1569 cells displayed an equivalent activity profile to trastuzumab in 3D cell culture in comparison to 2D monolayer cell culture. Furthermore, the results from this study also demonstrated the influence of the surrounding ECM microenvironment on the response of cells in 3D cultures to the Her2-targeted therapies by showing the combination of a β1-integrin inhibitor with each anti-Her2 agent generally enhanced the anti-tumor activity. 

3D modeling employing lrECM in the culture microenvironment has provided a unique tool for use in the elucidation of cellular signaling mediated by integrins. An early study demonstrated that inhibiting the function of β1-integrin in breast cancer cells (T4-2) cultured in 3D conditions triggered a reversion of these cultures to a non-malignant phenotype [18]. In the same study, α6- and β4-integrin function was inhibited in a 3D cultured non-malignant breast cancer cell line (S-1), and following treatment, these cultures exhibited features observed in malignant phenotype. Further research demonstrated that the phenotype of multiple breast cancer cells showed at least a partial morphological reversion to a normal tissue architecture when exposed to a number of specific inhibitors applied in combination or as single agents, for example, those targeting MAPK and/or β1-integrin [63]. Additionally, research was conducted into the influence of integrin binding on the formation of 3D structures. A synthetic hydrogel consisting of RGD binding sites was utilized, with results demonstrating enhanced growth of ovarian cancer cells upon integrin attachment to the substrate [64]. β1-integrin was also explored as a potential drug target utilizing 3D cell culture models situated in an lrECM-containing microenvironment. Blocking the function of β1-integrin in breast cancer cell (T4-2, MDA-MB-231, BT-474, MCF-7 and SKBR3) cultured as pre-formed 3D structures successfully inhibited the growth of these malignant cells [19] and enhanced the anti-cancer affects of breast cancer cells (MCF-7 and T4-2) following exposure to ionizing radiation [65]. 

The investigation of resistance against anti-Her2 therapeutics in breast cancer cell lines revealed that β1-integrin downstream signaling has a role in mediating this altered sensitivity [66,67]. β1‑integrin was also demonstrated to be protective against several anti-cancer agents in hepatoma cells [68]. Furthermore, attachment of cells to lrECM was shown to protect cells from apoptosis in ovarian cancer 3D cultures upon exposure to the PI3K/the mammalian target of rapamycin (mTOR) inhibitor, BEZ235 [20]. The altered drug sensitivity was attributed to the up-regulation of pathways specific to cellular survival. Targeting the pro-survival protein, Bcl-2, insulin growth factor type 1 receptor (IGF1R) or epidermal growth factor receptor (EGFR) in combination with BEZ235 abolished the resistance observed with matrix-attached cells. Therefore, the presence of ECM is an important factor when considering the efficacy of therapeutics in the *in vitro* 3D tumor microenvironment.

Together, these studies highlight the differing genetic profiles, protein expression and drug sensitivity, which are evident in cells cultured in the more physiologically relevant 3D cell culture models compared to traditional 2D cell culture models. These studies also emphasize the importance of using 3D cell cultures to complete mechanistic studies on current chemotherapeutics and novel drug candidates. An awareness of the differences, sometimes significant, between cells cultured in 2D and 3D is an important factor when considering which model to select for the screening of new molecular entities. The benefits of screening biologically active compounds against cells in 3D culture models is their ability to account for these changes e.g., the switch in PI3K pathway signaling to MAPK pathway signaling observed in 3D cancer cell cultures, but not in 2D monolayer cell cultures as mentioned above [42,57]. The challenge is to incorporate the essential elements of these models into early-stage drug discovery practices.

## 5. Utilizing 3D Tumor Models in Drug Discovery: Progress So Far

The development and use of 3D cell cultures in drug discovery is becoming more prevalent. At the present time, a collaboration of academic laboratories and pharmaceutical/biotechnology companies in Europe has been established to develop new, more relevant, *in vitro* models for drug discovery practices [69]. Numerous methodologies have been established for novel compound screening practices utilizing 3D cell culture systems in cancer, particularly within the last few years. These procedures have included both non-adherent 3D cell cultures (anchorage-independent) and 3D structures which adhere to a substrate (anchorage-dependent). The assays have not only been developed by pharmaceutical companies, but also academic laboratories (Table 1). Below, we describe the outcomes from published assays that have been established and utilized in the screening of either a library of compounds/clincally relevant drugs or a small panel of reference drugs. 

**Table 1:** Three-dimensional cell culture amenable assay technologies.

Assay Chemistry and Endpoint	Commercial Products	Reference
**Microscopy (object-based)**		
**Cell Viability**		
Live/dead cell staining assay	LIVE/DEAD^®^ Viability/Cytotoxicity Kit	[70]
Live cell staining assay	Calcein AM dye	[21]
Live/dead cell staining assay	Hoechst and Sytox Green dyes	[71]
**Invasiveness **		
Brightfield		[5,29]
**Spheroid Size Analysis**		
Brightfield		[5,29,72]
**Colony Count and Size**		
Qdots/Calcein AM	Qtracker^®^ 625 Cell Labeling Kit	[73]
**Architectural Disruption of 3D Cell Cultures**		
Live/dead cell staining assay	LIVE/DEAD^®^ Viability/Cytotoxicity Kit	[70]
**Plate Reader (whole-well)**		
**Cell Viability **		
Tetrazolium reduction assays (MTT, MTS)	CellTiter 96^®^ AQueous One Solution Cell Proliferation Assay (MTS)	[27]
Resazurin reduction assay	alamarBlue^®^ cell viability reagent, CellTiter-Blue^®^ Cell Viability Assay, Resazurin sodium salt	[8,21,74,75]
ATP measurement assay	CellTiter-Glo^®^ Luminescent Cell Viability Assay	[5,76]
Acidic phosphatase (APH) assay		[77,78]
**Apoptosis Assessment**		
ELISA (caspase-cleaved CK18 fragments)	M30 Apoptosense^®^ ELISA	[79]
**Epithelial-to-Mesenchymal Transition Related Protein Expression**		
Luminescent reporter protein		[78]

### 5.1. Anchorage-Dependent 3D Tumor Models Developed for Use in Drug Discovery Programmes

#### 5.1.1. Breast, Pancreatic and Ovarian Cancer

Numerous anchorage-dependent models for drug discovery have been developed. Recently, we developed miniaturized 3D cell culture assays utilizing small panels of both breast (MCF-7, MDA-MB-231 and BT-474) and pancreatic (Panc-1, AsPc-1 and BxPc-3) cancer cell lines, suitable for use in drug discovery programmes [21]. The assays established were based on 3D cellular structures situated on lrECM in a 384-well microtitre plate format, a configuration compatible with a range of liquid handling, imaging and multilabel plate reading equipment. A pilot screen was conducted using a small library of 741 clinically relevant drugs and compounds, initially measuring cellular activity in 2D monolayer cell cultures, followed by re-testing of active anti-cancer agents against cells in both 2D and 3D cell culture models. The outcomes included the identification of 10 drugs displaying anti-cancer activity on the above-mentioned cells, with one drug, an anti-parasitic (maduramicin ammonium) not previously reported as exhibiting cytotoxic activity against cancer cells. The remaining drugs that demonstrated anti-cancer activity against selected breast and pancreatic cancer cell lines in the screen included statins, an immunosuppressant, an iron chelator and a cardiac glyoside. The drugs identified and subsequently characterized in this study exhibited activity against cells in both 2D and 3D cell cultures, however, the cellular response to a number of these drugs was model-dependent.

An anchorage-dependent 3D cell culture model utilizing lrECM as a scaffold, an anchorage-independent model utilizing poly-HEMA to induce spheroid formation and a 2D monolayer cell culture model were employed to test a library of 102 drugs and compounds against the JIMT1 breast cancer cell line (over-expresses Her2) in a 384-well microtitre plate format [76]. Sixty-three drugs/compounds exhibited greater than a 30% reduction in cell viability in one or more of the above-mentioned models. However, a large difference in drug/compound activity was observed between cells cultured in these different models. For example, colchicine, a drug used for the treatment of gout, inhibited cells cultured in the 2D monolayer and the 3D lrECM format to a greater capacity than observed for cells cultured in the anchorage-independent model. Interestingly, the gene expression of cells cultured in the 3D lrECM model was shown to more closely resemble the *in vivo* situation.

A high-content approach to the quantitative assessment of 3D tumor models utilizing a Matrigel-based lrECM model was developed. In this study, pancreatic (Panc-1) and ovarian (NIH:OVCAR-5) cancer cell lines were cultured in 96-well microtitre plates and 5 clinically relevant cytotoxic compounds were used to validate the system [70]. The study looked at streamlining the methodology and analysis of high-content 3D cell culture models while maintaining the screening speed traditionally achieved in whole-well reporter assays. Multiple assessment endpoints were measured from the cultures exposed to treatments including cell viability-based on cytotoxicity and growth inhibition in addition to structure and size-dependent responses. The system presented can be incorporated into laboratories with standard imaging and computer equipment while producing multiple quantitative readouts.

#### 5.1.2. Prostate Cancer

Krausz *et al.* [72] recently published anchorage-dependent 3D cell culture assay methodology for prostate cancer, which is suitable for high-content screening (HCS) (96-well microtitre plate format). Initially, comparisons of various imaging platforms (MIAS-2^®^, IN Cell Analyzer 2000^®^ and an Opera^®^) and their respective analysis programs along with their in house tool, Plate-based High-Content Analysis Evaluation and Dynamic Reliability Assurance (Phaedra) were performed. There were no major differences reported between data obtained from the imaging and analysis of cells in 3D structures for all of the instruments utilized. Next, a 3D co-culture assay was developed consisting of bone marrow stromal cells with prostate (PC3-M) tumor cells situated in lrECM. Anti-cancer agents, including topoisomerase I, mitogen-activated protein kinase kinase (MEK-1/2), SRC kinase and histone deacetylase inhibitors were evaluated, with reproducible data acquired. In a separate study, more than 100 compounds were screened against prostate cancer PC3 and PC3-M cells in a 3D cell culture system which utilized an lrECM framework in addition to a corresponding 2D cell culture assay (384-well microtitre plate format) [29]. The compounds demonstrating anti-cancer activity on these cell lines were further tested on a panel of prostate cancer cell lines (EP156T, RWPE-1, DU145, LNCaP and 22rV1) in 3D cell culture. The findings from this study included the identification of compounds that targeted PI3K pathway signaling in cells. These compounds inhibited the invasive properties of tumor cells cultured in 3D, yet demonstrated reduced efficacy against cells in corresponding 2D monolayer cell culture. Alternatively, the majority of inhibitors targeting mTOR and IGF1R signal transduction were effective against malignant and non-malignant cell lines cultured as both monolayers and 3D structures. Thus, the target of the compound would appear to dictate whether the cellular response is dependent on the cell culture conditions utilized.

#### 5.1.3. Lung Cancer

Differences in the susceptibility of cells to anti-cancer agents in alternative cell culture systems has been reported for the lung cancer cell lines, A549 and H358 [27]. These cell lines were cultured as 3D structures within a collagen I matrix in a 96-well microtitre plate and in a corresponding 2D monolayer cell culture assay format. The outcomes from this study demonstrated that the cellular sensitivity to the anti-cancer agents was cell line-, drug- and culture method-dependent. For instance, in the A549 cell line, the potency of alimta, zactima, and gemcitabine were significantly higher, whilst paclitaxel, KU174, doxorubicin and vinorelbine were significantly lower in cells cultured in 3D in comparison to cells cultured in 2D. The activities of cisplatin, 17AAg and KU363 were comparable against cells cultured in both 2D and 3D assays. The presence of a collagen I framework therefore influences drug sensitivity, whether the effects be dependent on the 3D cellular architecture or ECM-to-cell signaling. 

Collagen I and lrECM have been utilized as support networks for the culture of 3D cellular structures, whether for mono-culture or co-culture, in microtitre plates including both 96- and 384-well formats for a wide range of cancers. Anchorage-dependent assays developed for use in drug discovery platforms have either been validated through the screening of biologically active drugs/compounds or a small panel of chemotherapeutic agents, with a substantial volume of results demonstrating the altered senstivity of cells to drugs/compounds when cultured in a 3D architecture with substrata.

### 5.2. Anchorage-Independent 3D Tumor Models Established for Evaluation of New Molecular Entities

#### 5.2.1. Brain, Breast and Oral Cancer

In addition to the anchorage-dependent 3D models, a range of anchorage-independent assays suitable for screening practices have also been developed. For instance, several different inhibitors including heat shock protein 90 (17-*N*-allylamino-17-demthoxy-geldanomycin; 17AAG), PI3K/mTOR (PI-103) and phospholipase C (CCT130234) were evaluated in numerous cancer cell lines (glioblastoma: U-87 MG, KNS42; breast cancer: MDA-MB-231 and oral squamous cell carcinoma: LICR-LON-HN4) utilizing an anchorage-independent 3D cell culture assay in a 96-well format [5]. Following exposure to the various inhibitors, cellular viability was demonstrated to be approximately equal to, or higher, when 2D monolayer cell culture was compared to 3D cell culture spheroids. This anchorage-independent assay methodology could also be extended to measure the migration and invasion of tumor cells upon treatment. Whilst these assays have not been utilized for the screening of novel compounds, the above-mentioned anti-cancer agents were used to exemplify the inhibitory actions of these therapeutics against cells on the different 3D cell culture platforms. 

In a study also utilizing the breast cancer cell line, MDA-MB-231, an epithelial-to-mesenchymal transistion (EMT) anchorage-independent spheroid model (96-well microtitre plate format) was developed by incorporating a luciferase reporter of EMT in cells [78]. Two-hundred and thirty compounds were screened and their activity against MDA-MB-231 cells determined in luminescence and cellular viability assays. Four compounds that selectively inhibited EMT were identified: mycalolide E and jaspamide, which both target F-actin; and isonaamidine B and papuamine, which have unknown modes of action. In a separate breast cancer study, a HCS assay was established in a 384-well microtitre plate format using the breast cancer cell line, TD74, cultured as MCTS to specifically detect compounds that target dormant cells located within the spheroid interior [71]. Following the screening of 1120 compounds, 9 respiratory chain inhibitors targeting these quiescent cells were identified. A unique 3D cell culture model allowed selective detection of the inhibition of EMT in conjunction with cellular viability and, in addition, 3D cell culture methodology facilitated the detection of inhibitors specifically targeting dormant cells.

#### 5.2.2. Pancreatic Cancer

A panel of pancreatic cell lines (AsPc-1, BxPc-3, Capan-1 and Panc-1) were utilized in an anchorage-independent assay developed into a 96-well microtitre plate using methylcellulose as a cell-repellant to stimulate spheroid formation [77]. Eleven compounds with a range of targets, for example, microtubulin, were examined. The outcomes included compounds (MT100, Allicin and AXP-107-11) which exhibited activity against cells grown in both 2D and 3D conditions. However, the majority of compounds (H107, CB5, CB7, CB13, 6-MP, 6-MPR, act16412 and GANT61) generally demonstrated modest activity against cells cultured in 2D, but only limited activity against cells grown in 3D culture. Thus, the cells cultured in this 3D model frequently demonstrated reduced sensitivity to a range of compounds when compared to the same cells cultured in 2D monolayer conditions. The differences in compound activity against cells in these culture systems may have been mediated by the more chemoresistant phenotype of spheroids observed in this study, for example, the increased amount of ECM proteins present in these 3D cell cultures. 

#### 5.2.3. Lung and Colon Cancer

A soft agar colony formation assay was established in a 384-well microtitre plate format for the lung cancer cell line HCC827 [75]. A library of 9600 compounds was tested against these cells cultured in both a 2D monolayer and 3D formats. The majority of active compounds (unknown targets) exhibited similar activity against cells irrespective of whether cultured in 2D or 3D conditions. This assay demonstrated the development of the first published soft agar assay in a 384-well microtitre plate format and the successful screening of a library of compounds. Horman *et al.* [73] adapted this soft agar colony formation assay to screen colon cancer cells (HCT116) which were cultured with and without normal colon fibroblasts (CCD-18Co). The 3D mono-culture assay was utilized in the testing of a library of 1528 compounds derived from natural sources. The criteria for selecting active compounds was at least a 60% reduction in growth of mono-cultured cells in 3D structures. The 83 active compounds were re-tested against cells in the 3D co-culture model, with a re-confirmation rate of approximately 50%. Thirty compounds were active against these HCT116 cells with an IC_50_ below 1 μM. Interestingly, a selection of compounds demonstrated increased activity against the colon cancer cells in comparison to the normal fibroblasts and, in addition, certain compounds inhibited the growth of only the cells tested in 3D cultures, not 2D monolayers. These results emphasize the importance of assessing compounds against cellular activity in 3D culture models, as active compounds may have remained undiscovered when applied to cells in standard 2D monolayer systems. 

An anchorage-independent 3D lung cancer model which utilized Algimatrix™ as a scaffold was developed for use in screening practices [74]. Lung cancer cells (H460, A549, H1650 and H1650 stem cells) in both 2D and 3D cell culture systems were tested against a small panel of chemotherapeutics including docetaxel, cisplatin, gemcitabine, 5-fluorouracil and camptothecin. The outcome of this study showed that cells cultured in 3D were more resistant (5–20 fold) across the entire panel of drugs when compared to cells cultured in 2D conditions, with the stem cell enriched culture exhibiting greater resistance than the parental cell line (H1650). These results show the use of a chemically defined scaffold to culture cells 3D and its potential for use in evaluating the activity of anti-cancer agents. 

The colon cancer cell line (HCT116) was also employed in an additional anchorage-independent 3D cell culture assay (96-well microtitre plate format) [79]. This assay was established to identify compounds that specifically trigger cellular apoptosis. A library containing a collection of 77 compounds and anti-cancer drugs were screened against these HCT116 cell line-containing spheroids, with numerous drugs/compounds shown to induce apoptosis. However, the ability of these drugs/compounds to instigate apoptosis was dependent on whether the colon cancer cells were cultured in 2D monolayers or 3D in culture. For instance, tamoxifen was demonstrated to trigger apoptosis in cells cultured in both types of models, but in contrast, cisplatin showed enhanced activity against cells in 2D culture in comparison to the same cells cultured in 3D. Thus, this research shows the development and assessment of an anchorage-independent spheroid assay for apoptosis endpoint detection and illustrates the capabilities of the assay with established anti-cancer agents. 

#### 5.2.4. Epidermoid Cancer

An epidermoid cancer cell line over-expressing mesothelin (A431.H9) was used in the development of a high-throughput hanging drop assay in a 384-well format, with cellular viability measured with alamarBlue [8]. The differing sensitivities of cell in 3D cell cultures were demonstrated utilizing the two drugs of tirapazamine (cellular inhibitor which causes DNA damage; demonstrates enhanced activity in the presence of hypoxia) and 5-fluorouracil (blocks the propagation of cells). Cells cultured in 3D exhibited resistance in comparison to the same cells cultured in 2D monolayers upon exposure to 5-fluorouracil. However, when tirapazamine was applied, the cells grown in 3D culture demonstrated increased sensitivity in contrast to those cultivated in 2D monolayer culture. These distinct responses were attributed to the altered properties of cellular activity in 3D conditions in comparison to that of cells in 2D monolayers. This study demonstrates the development and evaluation of a hanging drop assay which utilizes a 384 hanging drop array plate suitable for integration into HTS platforms.

Collectively, numerous 3D cell culture assays have been developed and represent progress towards recapitulating the complete tumor microenvironment in comparison to utilizing only traditional 2D monolayer cell culture models for the screening of drug candidates. The inclusion of certain tumor components in addition to cells cultured in 3D conditions has contributed to successfully identifying differences in the anti-cancer activity of compounds/drugs in selected screens. However, there is still a significant amount of research required to establish 3D cell culture assays that not only possess the essential elements of a tumor in a panel of cancer cell types, but are automated and analyzed in a high-throughput manner.

## 6. Towards More Complex Assays Suitable for Integration in Drug Discovery Programmes

Screening programs rely on various forms of technology for determining the anti-cancer activity of compounds. The bottleneck for incorporation of more complex model systems into future drug screening is not only in tissue engineering and developing improved materials that reproduce optimal *in vivo*-like biological conditions, but also with the ability to apply these approaches in a drug discovery setting. An increasing number of companies are developing and manufacturing substrates, assay plates and scaffolds for the formation of 3D structures (for details see [31,80]). The increase in commercial interest in 3D cell-based technologies also applies to hardware and software aimed at this evolving industry. 

To increase the throughput of compound screening in 3D cell culture, assays are required to be compatible with robotic systems including liquid handling technologies, which are generally in a 96- to 1536-well microtitre plate format. The use of these robotics allows accurate aspiration and dispensing of assay reagents and drug/compound in a timely manner. However, these liquid handling technologies, along with the data acquisition equipment, data analysis software packages and data storage/processing systems suitable for high-throughput applications have traditionally been expensive and not accessable to all laboratories. However, as access to high-throughput equipment such as liquid handling robotics and HCS platforms continues to become more viable, the integration of complex biological low-throughput models into formats suitable for the screening of compounds may be possible. 

In recent years technical advances in imaging based technology has allowed for HCS based assays to be used in a number of 3D applications. Only recently has the computing power required to acquire and analyze large Z-stack data sets (critical for 3D cell culture) become available as a mainstream commercial product. Software solutions for complex analysis of 3D data are now available both commercially and as open source (Imaris^®^, Volocity^®^, Fiji). Several of these programs consist of features which enable batch analysis of large data sets that are becoming readily accessible for most academic groups, and are relatively user friendly. In addition, various analysis protocols which focus on deconstructing elements of cells cultured in 3D, such as morphological profiles and biomarkers, designed for low-throughput and HCS experiments have been developed and published [72,81,82]. Technological advances such as these may not only allow a broader scope of models to be developed, but may further encourage the incorporation of 3D models in the search for anti-cancer drug candidates in the drug discovery pipeline.

Scientific advances have resulted in the development of novel 3D cell culture models. These advances are in the form of a multitude of approaches including microfluidics [83,84,85] and novel scaffold/hydrogel designs [86,87]. These models have the potential to reduce the cost of materials and allow integration of elements, such as perfusion, that are unable to be incorporated into other models. Characteristics such as nutrient, gas and drug diffusion rates, cell shear stresses and other microenvironmental conditions can be more accurately controlled in these systems [88]. This tissue engineering technology is particularly suitable for not only replicating tumor microenvironments but for high-throughput drug screening conditons.

Progress has been made in cell biology with the expansion of 3D cell culture models into 3D co-culture systems. 3D cancer co-culture models have been developed in both low-throughput [89,90,91,92] and high-throughput [5,72,73] formats. However, at the present time, only cancer cells along with one other cell type (e.g., fibroblasts) have been incorporated into models suitable in screening new compounds in a high-throughput manner. 3D cell cultures have the ability to recapitulate certain biological aspects of tumors, with the physical properties of the ECM playing a key role in addition to ECM-to-cell signaling [93]. The rigidity of the tumor microenvironment is frequently increased in comparison to normal tissues [94]. The increase in stiffness of the ECM surrounding tumor cells has been demonstrated to alter tissue structure and augment growth [95]. These mechanical forces within the *in vitro* 3D structure are model-dependent and are not generally taken into consideration when screening new molecular entities.

In addition to the application of 3D cell cultures to advance *in vitro* cancer models, there are alternative model systems that possess complexity between *in vitro* 2D monolayer and *in vivo* cancer models. These include the utilization of *ex vivo* approaches such as tissue slices [96] and Hi-Spot/OrganDots (tissue extraction, separation and re-aggregation into structures that exhibit similarities to the tissue in question on a membrane substrate) [97,98]. The limitations of using explanted tissues include inconsistency of tissue sources and the restricted supply of rodent/human tissue [98]. Whilst these model systems are applicable for use within small-scale studies evaluating novel molecular entities, they are currently not suitable for incorporation into early-stage drug discovery practices that require examination of a range of potential anti-cancer agents in a high-throughput manner.

Personalized medicine based on patient-derived cells has the possiblity to significantly enhance anti-cancer treatment for targeted molecular therapies. However, the application of patient-derived tumor models has been mostly limited to low-throughput pre-clinical testing to date [99,100]. Commercial institutes, such as Oncotest, have developed 3D assays based on indirect patient-derived samples, which have been expanded through the utilization of mouse xenograft models. Culturing of primary cells from patients and standardization for use in drug screening programs is currently a technical challenge. However, a 3D tumor model system based on multiple patient-derived cells to be incorporated into early-stage drug discovery may be the future of personalized drug development programmes.

There is increasing information available detailing the advantages and disadvantages of using 3D tumor models in early-stage drug discovery practices. However, much of the signaling changes and drug efficacy data remain unvalidated in an *in vivo* model. There are numerous 3D cell culture models developed for examination of drugs/novel compounds, but which model is the most representive of a tumor for a particular cancer? In addition, which cell types from the tumor microenvironment are to be included in 3D cancer co-culture models? It remains to be seen which model/s will prove the most useful in drug discovery practices.

Another approach that may be applicable to the future of advanced screening models is the standardization of 3D tumor models. For instance, what is the optimal drug exposure period? How large (cell number, diameter) should 3D structures be prior to drug addition? Also, which model is the most appropriate for each particular cancer? There are specific conditions in certain cancers required for enabling development of the most *in vivo*-like model possible. For example, ovarian cancer requires mesothelial cells which line peritoneal surfaces (the most common location for metastatic growth) to better represent the metastatic disease [101]. These conditions are unique to ovarian cancer and would therefore not be applicable for use in advanced *in vitro* models for other cancers. The question is then raised regarding how effectively 3D cell culture models can really be standarized over a large range of cancers, which does not even begin take into account all the different stages of disease within each type of cancer and the diverse sites of metastases. Would it be best to create models as close to the tumor microenvironment as possible and set out to standardize other parameters such as length of cellular exposure to drug and the size of the 3D cultures? Or, perhaps attempt to develop a universal 3D cell culture method that encompasses the key elements of all cancers? Further studies to determine the most representative advanced 3D tumor microenvironment models for each type of cancer may allow researchers to determine which cancers types can and cannot be standarized for use in a specific type of model. 

## 7. Conclusions

A clearer understanding of the complex mechanisms influencing the mode of action and efficacy of cancer therapeutics is essential to move closer towards the goal of eradicating cancer cells in the patient. Research into new 3D tumor models that more closely represent the tumor microenvironment in systems that are scalable to meet the requirements of screening practices is underway, with notable progress published recently. However, there currently lacks a consensus with respect to which model reflects the nature of the various tumor types and therefore should be utilized for screening practices. In addition, the current 3D cell culture models recapitulate certain elements of the cancer setting, however, numerous components are excluded for each type of 3D cell culture model. Further advances in technology, personalized medicine and model development will no doubt overcome these obstacles. 
